# Touch and manual action in chemotherapy-induced peripheral neuropathy: a mixed-methods study

**DOI:** 10.1038/s41598-026-46039-2

**Published:** 2026-03-29

**Authors:** Roberta D. Roberts, Winnie Chua, Ali Khatibi, Nicholas P. Holmes, Claire Palles, Racha Kussaibati, Alan M. Wing

**Affiliations:** 1 School of Psychology, University of Birmingham, Edgbaston, Birmingham, UK; 2 Department of Psychology, University of Bath, Bath, BA2 7AY UK; 3 School of Sport, Exercise and Rehabilitation Sciences, University of Birmingham, Edgbaston, Birmingham, UK; 4 Department of Cancer and Genomic Sciences, University of Birmingham, Edgbaston, Birmingham, UK; 5 The National Institute for Health and Care Research (NIHR) Birmingham Biomedical Research Centre, Birmingham, UK; 6 Department of Oncology, Heartlands Hospital, University Hospitals Birmingham NHS Foundation Trust, Birmingham, UK

**Keywords:** Chemotherapy induced peripheral neuropathy, Sensorimotor, Touch impairment, Manual dexterity, Patient reported outcomes (PROMS), Questionnaires, Focus group, Deductive quantitative analysis, Health care, Medical research, Neurology, Neuroscience

## Abstract

Chemotherapy induced peripheral neuropathy (CIPN) is a frequent side effect of a number of chemotherapeutic agents which are widely used in the treatment of common cancers. Sensory symptoms primarily affect the fingers and toes and include numbness, tingling and pain. However, there is limited understanding of how these touch impairments may impact manipulation skills. Questionnaire and focus group methods were used to describe the experience of CIPN side effects impacting manual activities in 25 self-selected participants recruited from a cancer charity web site advertisement. Participants’ responses demonstrated varying degrees of impact of touch impairment associated with CIPN on the performance of manual activities involving bimanual, unimanual and directed touch. Examination of the components of the affected activities, together with participants’ reports of alterations in their experience of touch, were used to generate theory-informed hypotheses about why some manual tasks are more affected than others. In future research it is proposed that quantitative objective measures should be used to complement patient reported outcome measures in evaluating the mechanisms underlying issues in sensory motor control of manual activities caused by the effects of CIPN.

**Supplementary Information:**

The online version contains supplementary material available at 10.1038/s41598-026-46039-2.

## Introduction

Chemotherapy induced peripheral neuropathy (CIPN) is a common side effect of several chemotherapeutic agents including taxanes and platinum analogues, both of which are widely used in the treatment of common cancers such as breast, prostate, lung and gastrointestinal malignancies. CIPN affects upwards of 60% of patients^1^. Sensory symptoms primarily affect fingers and toes (stocking and glove syndrome) and include numbness, tingling (paraesthesia), unpleasant sensation (dysaesthesia) and pain following stimuli that do not normally cause pain (allodynia). These symptoms tend to persist after termination of chemotherapy, and in some cases worsen for a period of time (coasting) and can affect activities of daily living and severely compromise patients’ quality of life. Various physiological mechanisms for CIPN have been proposed, but it is generally agreed that changes in synaptic or nerve conduction properties disrupt afferents between cutaneous (tactile) mechanoreceptors and the spinal cord^1^. The onset of CIPN may lead to treatment alterations including dose reduction and early cessation of chemotherapy which can ultimately lead to poorer outcomes.

While sensory aspects of CIPN have been investigated, and impact on quality of life and daily living measured^2–4^, the interaction between altered sensation and changes in manual behaviour in CIPN is less understood. In the present study we use patient-reported outcome measures (PROMs), supplemented by focus groups, to examine the reported experience of CIPN in 25 individuals, in order to identify manual activities of daily living that are affected, and to determine how participants cope with the impact of CIPN. We take a deductive qualitative analysis (DQA) approach^5^ and interpret these reports within current understanding of sensorimotor control including biomechanical, neurophysiological and psychological factors. Our aim is to facilitate the development of principles to explain a seemingly diverse set of tasks impacted by CIPN. We also ask whether future CIPN assessment might incorporate objective tests that bridge the gap between measures of elemental passive touch perception and their limited scope in describing CIPN impairment^6^, such as pressure and vibration detection, and complex, action-oriented, activities such as writing and buttoning skills.

Our approach has potential benefits for advancing theory in sensorimotor control, however we see further possible benefits. Based on observations of clinician-patient consultations and semi-structured interviews, Tanay and colleagues^7^ suggested that improved understanding of the effects of CIPN would benefit both clinical and patient management of the disorder, as well as the overall patient experience of living with CIPN. This point is underscored by the conclusion, based on a meta-analysis, of Amarelo et al.^8^, of the need for patient-centred strategies for coping with the effects of CIPN. The present work aims to contribute to understanding the effects of impaired touch on actions of daily living to support the future development of a framework for use by patients, clinicians and therapists in understanding and managing CIPN in daily life.

## Materials and methods

The study comprised two parts. The first involved questionnaires completed by a self-selected sample of people with lived experience of CIPN. The second part involved two focus groups drawn from a subset of those who completed the questionnaires. Ethical approval for the research was obtained from the University of Birmingham Science, Technology, Engineering and Mathematics Ethical Review Committee.

### Participants

The questionnaires were completed by 25 adults (including author AMW) with lived experience of CIPN. Their ages ranged from 46 to 75 years (mean 62.5 ± 8.2); 8 were male and 17 were female. They were recruited through an advertisement on the Cancer Research UK website.

### Questionnaires

Participants completed an online version of 3 questionnaires, accessed in Spring 2023. The first was the European Organization for Research and Treatment of Cancer Quality of Life Questionnaire EORTC CIPN20, abbreviated here to QLQ^9^. QLQ, which was favourably reviewed by Li et al.^10^, consists of 20 items, developed using European EORTC Quality of Life Group guidelines, to measure patients’ experience of symptoms and functional limitations related to CIPN. Item inclusion in the questionnaire reflects, amongst other factors, the prevalence, relevance and importance to patients of the issue described. The 20 items in QLQ consist of separate questions for hand and foot relating both to CIPN symptoms experienced and a set of 3 manual activities affected (difficulty holding a pen which impacts writing, difficulty manipulating small objects with the fingers such as buttoning clothes, difficulty opening a jar), in the previous 7 days. The second questionnaire was the Patient Neurotoxicity Questionnaire PNQ^11^, also reviewed by Li et al.^10^, which has questions covering the previous 24 h in terms of symptoms across upper and lower limb and a longer checklist of activities affected including the 3 in the QLQ. The third questionnaire, which was developed for the study, is referred to as the University of Birmingham Questionnaire (UBQ). UBQ is more focused on numbness and tingling than QLQ and PNQ but also more open-ended in seeking responses to activities affected, coping strategies and extending the relevant timescale for reporting of symptoms back to the onset of CIPN. Together, these questionnaires were used to characterise participants’ CIPN experience from its onset up to the time of completing the questionnaires. UBQ details and the distribution of responses to all questionnaires, can be found in the Supplementary Materials.

### Focus groups

Participants who indicated their interest while completing UBQ were invited to take part in an online focus group. Two focus groups of five participants were held in separate video conference (Zoom) sessions (A, B), each led by two of the authors (RDR, AMW) acting as facilitators and lasting one hour. The session began with a brief review of the questionnaires that the participants had completed 3 months previously, and a short presentation on neural pathways for touch based on the work of Witney and colleagues^12^. The facilitators then led the discussion of CIPN effects on the manual activities explored in the QLQ questionnaire by asking participants to write their name in pencil on a piece of paper and fasten a button on a shirt, items they had been asked to bring to the session. It is worth noting that more than half of the questionnaire respondents had reported difficulty carrying out these manual tasks. This is described further in the Results. During the focus group discussion, participants were invited to describe problems experienced in these and other manual activities and discuss strategies invoked to compensate for the impact of CIPN. The session concluded with discussion of sensory experiences of CIPN on the feet and how information reviewed in the focus group might be useful for others experiencing CIPN.

### Analysis

We took a DQA approach in which predefined themes originating in sensorimotor control theory were picked up with QLQ questionnaire items addressing manual symptoms (tingling, numbness, pain, cramps) and actions (writing, buttoning, opening a jar), and used to draw together the focus group discussions with the aim of theory refinement rather than inductive theme discovery^5^. As a check against possible author biases, the results, in preliminary form, were made available, first as a draft paper emailed to all participants who provided their email for this purpose (*N* = 19), then in open access form (doi: 10.1101/2025.03.05.25323401) with an email link and an invitation to make comments if desired. Of these participants, approximately half (*N* = 10) acknowledged receipt and half of these (*N* = 4) expressed agreement with and expanded upon aspects of the summary. None indicated any disagreement with the characterisation of participants’ views.

## Results

In our presentation of the results, we first summarise participant demographics and then report CIPN effects (in terms of both questionnaire responses and focus group statements) on hand sensation, on writing and buttoning activities, and on other manual activities. In some instances, the questionnaire responses of a subset of participants are presented in terms of averages over responses to individual questions and across grades of response.

The focus group reporting is shown in tables with the sequence of columns indicating focus group (A or B), participant study ID, the time stamp (minutes, seconds) in the recording and participants’ statements. The statements are reproduced verbatim except for omissions of asides (…) and italicised parenthetical comments inserted by the authors. There is no rephrasing, and the original ordering is preserved as it represents raw data. It is grouped by themes agreed by the authors and confirmed with the participants. This conforms to standards suggested by Anderson^13^.

### Demographics

Based on the 25 participants’ responses to UBQ item 3, 68% (*N* = 17) experienced numbness and tingling affecting the hands and the feet, 24% (*N* = 6) just the hands and 8% (*N* = 2) just the feet. The last two participants were excluded from the following analyses as these focused on the hand and the performance of manual activities. The demographics for the 23 participants are shown in Table 1.

**Table 1:** Participant demographic details.

Demographic Information	
Measure	Mean (SD) or number of participants
Age and range	62.5 (8.2), 46–75 years
Female	15
Right handed	20
CIPN onset during chemotherapy	17
Years since completing chemotherapy (SD)	4.3 (3.1) years
Number with CIPN improving	5

Chemotherapy information was available for 15 of the 23 participants. Of these, 10 received taxanes, 8 platinum compounds and 5 other agents, including Bortezomib, Lenvatinib, Pertuzumab, and Cyclophosphamide.

Table 2 reproduces statements from the two focus groups elaborating on the sensory experience of the hand. The statements relate to numbness, tingling and to pain. Comments under numbness and tingling include reductions in CIPN symptoms. Under the pain heading, statements include reference to pain interfering with tasks in which fingertip pressure is required.

**Table 2:** Focus group statements relating to sensory experience: hand numbness and tingling and pain. Focus group statements about sensory experience of the feet are provided in Supplementary Materials Table S5.

Group	ID	Time	Statement
			Numbness, tingling
A	25	27:55	So I now, I really only have any loss of feeling right in my fingertips right at the very end of my fingers. So my feeling has improved
B	26	14:18	Sometimes in the mornings I wake up, and I do this (*wiggles fingers*) to get them. I feel as if I’ve slept on them, even though I haven’t slept on them, you know, because wake up with the pain and tingling, and I can’t feel anything
B	11	16:30	I had the tingle in my hands for a good 6 months after the chemo and they were actually trying to work out … a better way to give me the chemo, so that I would have less problems with my hands … because I use my hands to see with (*participant 11 is blind*). In the end they stayed with the normal once every 3 weeks, but once, once the tingling … started, it just stayed
			Pain
A	19	34:03	There’s an interference, isn’t it? I think we’re saying that there’s some pain you can discriminate well enough. But now there’s a pain element which is when you press
A	25	39:06	Even now (*5 years on*), because the room is quite cool my fingertips are painful and so to take anything I know I’m touching because of the pain, more so than the sensation of touching
A	19	56:54	I’m wearing gloves in the morning, and also for the discomfort. If I was to touch something even just hooking the dog’s lead on and off, trying to use the clasp and things like that can be uncomfortable
B	10	03:59	I’ve got … few problems with my hands … the only thing I can’t do is play the guitar because of the pressure on the very tips of my fingers, and I just can’t do it now. … yes, it’s pain
B	11	04:19	I find it painful to read very new Braille for a long time because when it’s … it’s sharper. and so … I could only read it for so long, and then it would get painful, whereas if it was older, Braille is duller … it was easier to read

### Writing and buttoning activities affected by CIPN

More than half of participants reported that one or more of the three hand activities in QLQ were affected by CIPN (Table 3). They were (i) holding a pen, which made writing difficult, (ii) manipulating small objects with the fingers, for example, fastening small buttons, and (iii) opening a jar or bottle.

**Table 3:** Responses to hand-related QLQ questions for participants who indicated numbness and tingling due to CIPN affecting the hands in UBQ item 3 (*N* = 23). Responses to QLQ questions indicate percentage of participants responding with ratings greater than 1 for QLQ items 1 (tingling), 3 (numbness), 5 (pain), 7 (cramp) and items 11 (pen holding), 12 (button fastening), and 13 (jar opening). The distribution of gradings for all questions in the UBQ, QLQ and PNQ questionnaires across all 25 participants can be found in Tables S1–S3 in the Supplementary Materials.

EORTC CIPN20 Questionnaire (QLQ)—hand sensation and dexterity questions	
Measure	Percentage (number of participants)
Tingling in fingers or hands	74% (17)
Numbness in fingers or hands	65% (15)
Shooting or burning pain in fingers or hands	30% (7)
Cramps in hands	30% (7)
Difficulty holding a pen, impacting writing	52% (12)
Difficulty manipulating small objects with fingers	78% (18)
Difficulty opening a jar or bottle due to hand weakness	57% (13)

The pen holding and button fastening tasks were included in the focus group discussion, and associated statements are summarised in Table 4. With both writing and buttoning, subthemes were related to problems in holding and strategies to get a good grip. A further subtheme emerged for buttoning which was difficulty exerting sufficient pressure when buttoning up clothes.

**Table 4:** Focus group statements relating to hand activities—writing and doing up buttons.

Group	ID	Time	Statement
			Writing
A	30	25:03	It was quite tricky for me to concentrate on holding my pen properly and being able to write so I could understand what I’d actually written… prior to my chemo, I never had any trouble with my writing at all
A	25	28:88	I found it difficult to write… and I thought that was a brain issue and a confidence issue… and I switched to a thicker pencil… perfect for me…It’s got ridges in this section and I can feel the ridges… preferred implement for writing… because I can grip it better
A	23	31:16	My hand tends to slip little bit more. I tend to grip that more so I think a thicker pen might be easier to write with… my grip is not as good as it used to be
			Buttons
A	9	43:03	Sometimes it can be really quite difficult to get the buttons, because it’s a thicker material, … it is two-handed, and it’s … getting it quite in the right place too, and getting the pressure
A	25	44:24	What I do is to use kind of the pad of my finger rather than my finger tip to get a difficult button through a hole. I’m doing it now. I feel with the pad of my finger. I’m feeling the surface and the size of the button … because I can’t feel at the end. … And then what I was doing was actually then tipping the button on its side and using my fingernail … at one time during chemo and straight after, I couldn’t really do buttons up
A	30	46:01	I do find sometimes that it’s quite difficult to do the button up on my shorts or my trousers… I don’t recall having any trouble before, whereas I do now, particularly as I say, if my hands are cold
A	19	48:48	it took a wee bit longer, and it was a bit more awkward when you were doing like buttons down the front of your shirt. … But then I really did have problems with trying to do the cuffs, and it’s a single handed action. … and generally material is a bit thicker as well, and maybe the button hole is slightly tighter
A	23	49:29	I noticed it more when my hands are cold … So I do think there is a thing about blood flow or something. … affect your pressure and doing the buttons and that type of thing
B	22	07:26	I do use a buttonhole device to get dressed or alternatively buy clothing with stud or zip fasteners

### Other manual activities affected by CIPN

Table 5 summarises the activities affected by CIPN (and associated percentages of respondents affected) as captured by PNQ item 3 for the 14 participants who responded to this question. The results complement those for QLQ and highlight difficulty with buttons (plus other 2-handed manipulation activities involving buckles, shoelaces, jewellery and sewing). Difficulty in holding a pen for writing and using eating implements, all examples of single-handed tool use, also feature, but with lower proportions of respondents affected. Directed fingertip touch including using a touchscreen, phone, or keyboard constitutes another group of affected activities, again with lower proportions affected.

**Table 5:** Percentage of responses (total *N* = 14) to PNQ item 3 indicating problems with manual activities involving bimanual manipulation (doing up buttons, buckles, lacing shoes, fastening jewellery, sewing), One-hand tool use (writing, using eating implements), and directed touch (touchscreen, using phone, keyboard) activities. The responses to PNQ non-dexterity items, can be found in Table S3 of the Supplementary Materials.

Class	Activity				
Bimanual	Button	Buckle	Shoe laces	Jewellery	Sewing
Percentage (N)	79% (11)	50% (7)	43% (6)	50% (7)	29% (4)
One-hand	Write	Fork	Knife	Spoon	
Percentage (N)	36% (5)	29% (4)	29% (4)	21% (3)	
Directed	Touchscreen	Phone	Keyboard		
Percentage (N)	43% (6)	29% (4)	21% (3)		

Further insight into issues arising with manual activities comes from responses to UBQ item 5, “How does numbness/tingling affect activities?” Responses are given as a percentage of the 23 participants who responded to this question. Impaired dexterity (in picking up and manipulating items) (48%; *N* = 11) and dropping items due to misjudged grip (30%; *N* = 7) was described by between half and a third of the participants. In their responses the participants often alluded to loss of sensation, especially at the fingertips. Difficulty with achieving sufficient grip for both small (e.g., buttons) and large (e.g., jars) items was reported in 17% (*N* = 4) of cases. Some respondents attributed this to reduced finger strength. Several participants also reported slowed responses to temperature resulting in burns (13%; *N* = 3) and painful sensitivity to cold (9%; *N* = 2).

One third of participants (34%, *N* = 8) provided responses to UBQ item 7, “What helps reduce feeling of numbness and tingling?” and UBQ item 8 “What strategies help reduce impact of numbness and tingling on your activities?” In terms of reducing numbness, the suggestions they provided were keeping hands warm (wearing gloves) (63%; *N* = 5) and rubbing the skin (38%; *N* = 3). Strategies to help activities (25%; *N* = 2) involved using an aid (a jar opener or button hook helper), distraction or ignoring changes in sensation (25%; *N* = 2), and one each advised using muscle memory, moving slower, improving feel by using thicker wool (in knitting), and asking someone else to perform the task.

Various activities of daily living, ranging from washing dishes to knitting, were brought up in the focus groups (Table 6). Experiences included poor grip, especially with cold hands, shown under the theme of grip, and a feeling of clumsiness in fine manipulation, shown under the theme of dexterity, in Table 6.

**Table 6:** Focus group statements relating to grip failure and dexterity in other tasks involving the hands.

Group	ID	Time	Statement
			Grip failure
A	25	38:08	my husband said we needed to take out a bank loan for the amount of crockery that I dropped. Yes, I dropped a lot of things early on it doesn’t happen now thankfully
A	30	39:55	When I come inside I have to wait for ages for my hands to warm up, … to be able to be able to grab the shoe laces properly … I can’t do up my shoes if my hands are cold. Or I do them up and the laces come undone immediately
A	30	47:09	if I’m just trying to open a plastic box … for some reason, when my hands are cold, I just can’t get enough pressure in them to open the box properly. I fumble with it
B	26	14:43	I have dropped things before now because they (*fingers*) are numb
			Dexterity
A	9	43:59	(*when*) I’m reading the paper, … separating pieces of paper much more difficult, even though I’m you know, I’m relatively recovered
B	11	02:03	trying to do some fine knitting, and I was trying to count the stitches. I could hold the knitting needles, but I could not differentiate between the fine stitches with my fingertip (*participant 11 is blind*) … changed to heavier wools to “see” what I was doing
B	10	10:28	Initially, after the chemotherapy I did find my hands a little bit clumsy, but that quickly went away, and I have absolutely no problem with fine detail. I would say it was (over a period of) a few weeks, couple of months … it was really just when I tried to do something small that required a bit of manipulation that I found my fingers a bit clumsy

### Sensorimotor control interpretation of touch impacts

**Fig. 1:**
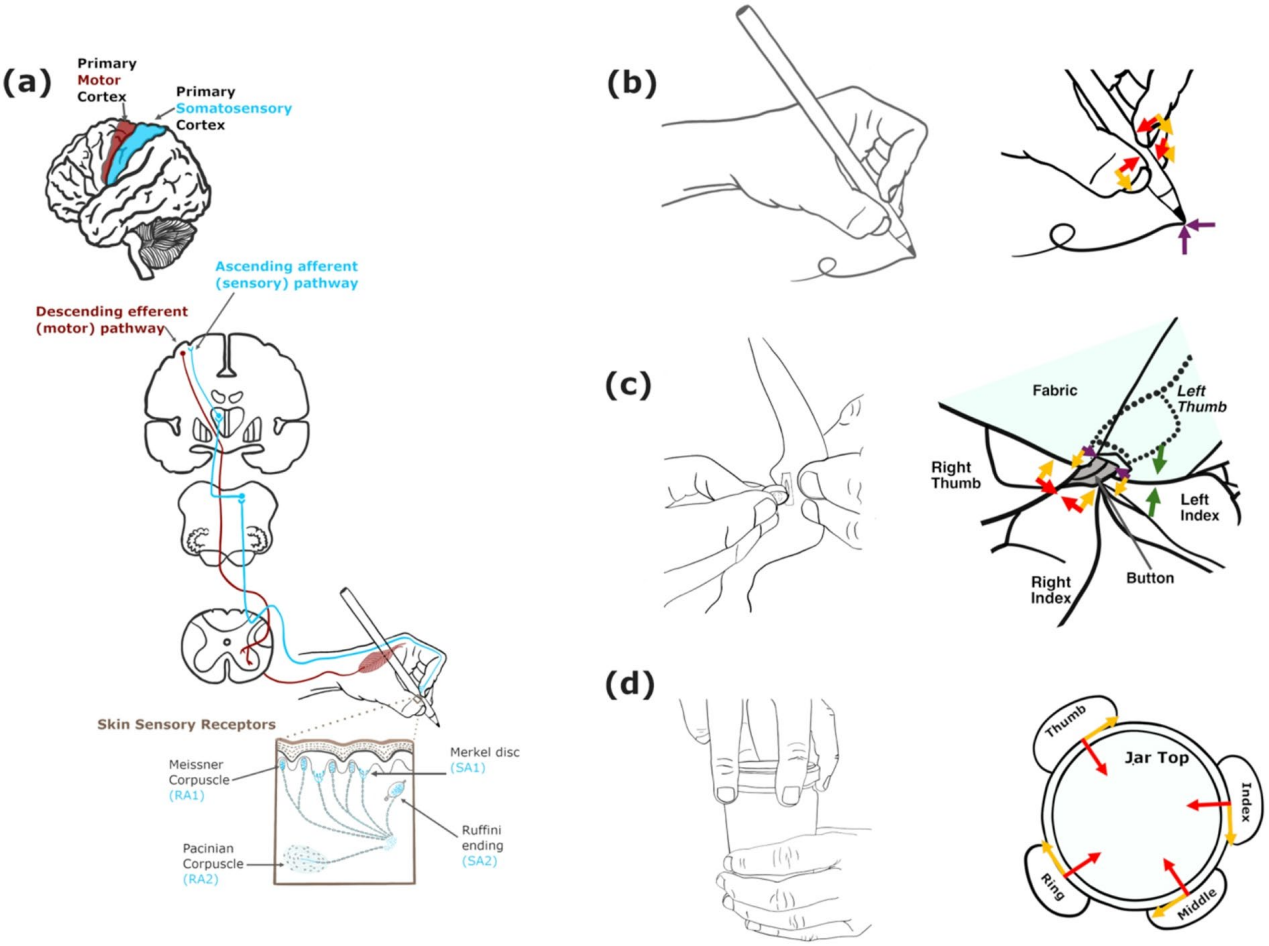
Grip and load forces in manipulation tasks: (**a**) Sensory motor pathways involved in manipulation (based on^12^). (**b**) Handwriting. In writing, the thumb, middle and index finger form a tripod. Grip force normal (shown by red arrows) to the pen surface develops friction between the pen and digits allowing tangential force (yellow arrows) to press the pen down on the paper without the digits slipping. Reactive forces to the pen tip, normal and tangential to the paper surface, are shown as purple arrows. Imbalance of the grip forces allows the pen tip to be pushed across the paper. (**c**) Buttoning a shirt. The top-down view shows the right-hand thumb and index finger grip force normal to the button surfaces (shown by red arrows) develop friction allowing tangential force (yellow arrows) to push the button into the buttonhole without the digits slipping. Purple arrows here show reactive forces from the buttonhole) which has been opened using a pinch grip (normal forces shown by green arrows) pull of the material with the thumb and index of the left hand. (**d**) Opening a jar. The thumb, index, middle and ring finger grasp and attempt to rotate the lid on the jar held by the other hand. Each digit exerts a normal force into the centre of the lid (shown by red arrows) which generates a friction to resist slipping due to the turning force (shown by yellow arrows) at each digit tangential to the lid. Tactile feedback from the multiple contact points is key to controlling grip and load forces needed to achieve these tasks.

Three tasks, using a pen, fastening buttons and opening jars, were singled out as problematic by QLQ and explored in the focus groups. What features, from the perspective of sensorimotor control, do these hand activities share and how do they differ? The activities involve locating (initially with vision, then guided by tactile contact) and taking hold of an object (or material) between the tips or pads of the finger (or fingers) and opposed thumb. What contact forces are involved in taking hold, and how is tactile feedback used to regulate the forces? Sufficient grip force, directed into the object (buttonhole, pen, or jar top), is needed to keep stable contact and avoid the fingers slipping across (or even losing contact with) the object while applying force along the object surface. This latter tangential force causes the object to move against frictional resistance provided by a second separate object (button), or a connected object (jar) or surface (writing paper), held by the other hand (Fig. 1). Such frictional resistance creates pressure and vibration at the contacting skin, providing sensory cues about the ongoing action and material properties of the object, on the basis of which motor commands to the muscles may be adjusted (Fig. 1a).

The touch feedback generated in each of the three activities can be used in developing sufficient grip force to provide frictional resistance to the load forces involved in manipulation, which tend to cause the grasped object to slip. However, these manipulation forces must be more accurately directed spatially in locating and inserting the button in the buttonhole and in shaping letters in writing compared to developing twist force (torque) between the all-digit grasp on the lid and power grip (palm and fingers together opposed by the thumb) on the jar. The contact in buttoning is more with the tips of the digits and involves both hands compared to finger pad manipulation with one hand in writing. Taking the requirement for two hand manipulation with CIPN involvement, particularly of the fingertips, may be the reason that buttoning was found to be more frequently affected than writing. In the case of jar opening, the forces are higher than the other two tasks which may be a significant factor in the difficulty associated with CIPN, especially if it is combined with dry skin, which is commonly experienced after chemotherapy^14^. Dry skin reduces friction and therefore increases the risk of the fingers slipping on the surface. This difficulty is compounded by CIPN effects on afferent fibres making it harder to detect slipping sensations which delays corrective action. In addition, it is important to note some participants commented that pain during forceful contact, especially when the hands were cold, is not only aversive but can mask discriminative touch needed for the feedback control of manipulation.

## Discussion

The present study explored patients’ experiences of, and strategies for, coping with touch and action changes following chemotherapy treatment. We relate such deficits to mechanisms of sensorimotor control with a view to improving understanding and impacting management of CIPN by clinicians, therapists and patients. This is consistent with recent questionnaire-based research^15^ and a qualitative meta-synthesis^8^ indicating a need for better understanding of the effects of CIPN.

The present study included 25 participants treated on average 4.3 (SD = 3.1) years previously with chemotherapy that induced CIPN. Twenty-three of the participants reported symptoms affecting the hands. This group was the focus of the current study. Of these participants more than half reported that their symptoms of numbness and tingling were not changing or were getting worse. Persistent or worsening CIPN symptoms over similar durations, or longer, have previously been reported^16–18^. The novelty of the present study is the way in which detailed descriptions obtained from the questionnaires and focus groups about difficulties in performing three particular tasks, using a pen, fastening buttons and opening jars, were given a sensorimotor control interpretation.

The impact of CIPN on other manual activities was picked up by the PNQ. The activities identified fell into three groups. The first group, affecting higher proportions of participants, was bimanual manipulation (doing up buttons, buckles, lacing shoes, fastening jewellery, and sewing). The sensorimotor control challenges for these activities will be seen as overlapping those discussed above for the QLQ buttoning task. The last two activities in this group, involving jewellery and sewing, might be seen as being easier in that they require lower forces than the rest of the tasks in the group. However, this easing of difficulty is likely to be offset by the extra dexterity required with reduced tactile feedback due to the smallness of the items being gripped which, in turn, necessitates using the very tips of the digits where the loss of tactile sensation is more pronounced. The second and third PNQ activity groups were one-hand tool use (writing, using eating implements), and directed touch (using touchscreen, phone, or keyboard), which involved lower proportions of participants. While sensorimotor control issues in one-hand tool use may be seen as similar to those in writing, the nature of difficulties in the third PNQ group is less clear. One possibility is that they relate to loss of touch feedback needed to register button contact and depressing the button.

Further insight into the effects of CIPN on sensorimotor control of manual activities comes from the responses to UBQ which included impaired dexterity in picking up small items, as well as dropping items due to misjudged grip. Both are consistent with reduced tactile feedback due to CIPN. In the focus groups, issues with grip that were raised included dropping crockery due to numbness and handling sheets of paper, for example, turning pages. Turning pages requires the tips of the fingers to gently rub and catch against the paper edge, which is likely to be a particular difficulty in CIPN as tactile feedback from the fingertips is especially reduced.

One participant’s suggestion for coping with touch impairment in handling objects was to move more slowly. From the perspective of sensorimotor control, this would be expected to help by reducing acceleration, which reduces the likelihood of a slip, and also by giving more time for processing of tactile feedback to achieve corrective increases in grip and avoid dropping the object. In the case of writing, enlarged or ridged pen grip surfaces were reported to be helpful. This may be because the increased contact area and the ridges improved tactile feedback. In addition, both may result in increased friction with the skin and so contribute greater resistance to slip. Other participant suggestions for reducing CIPN impact on manual activities included ignoring abnormal touch sensation and drawing more on muscle memory, both of which may change action control from “closed” (with feedback) to “open” loop (no feedback) or, possibly, pass feedback control of the action from touch to vision. Future research to evaluate the efficacy of these strategies is warranted.

Several participants reported problems with manual activities under the heading of general clumsiness. This suggests they were experiencing incoordination of finger movements relative to the object being manipulated. It would be useful to follow up on this and explore whether such incoordination arises from muscle weakness or reduced tactile feedback. A similar issue was brought up in the focus groups, where reference was made to reduced finger strength in opening a box and to poor grip on shoelaces, in both cases with cold fingers. Again, it is not clear whether this was due to impaired tactile feedback or muscle weakness. It is also possible that chemotherapy side effects of dry skin (reducing friction between digits and object surfaces, so requiring higher grip force to prevent slipping), and fingernail damage (impairing manipulation with the very tips of the fingers) play a role. Another possibility is that there are deficiencies in proprioceptive input from muscle receptors, and this could also contribute to movement problems in CIPN. Deficits in proprioceptive guided control of movement including both upper and lower limbs, have been reported in CIPN^19^ and it may be that proprioceptive deficits underlie reports of weakness, noted in the present study. Further research on these issues is merited.

### Limitations

The present findings involved in-depth exploration of a group of cancer patients’ experience of CIPN. However, this work was based on a relatively small number of self-selected participants, which could have introduced biases in sample composition, for example related to the recruitment source. Additionally, CIPN was assessed at a single time point, asking participants to report both recent experiences (QLQ and PNQ) and those from years past (UBQ, focus groups), with the latter potentially subject to recall biases. While it may be assumed that the findings will generalise to people with similar clinical and demographic characteristics, further research is needed, for example, to examine generalisability to other contexts. Such work should expand on the present research, for instance, collecting data on CIPN experience with repeated measures in a longitudinal design, and gathering more detailed demographic data such as those regarding ethnicity, tumour type and treatment specifics and durations.

The present study did not include age-matched controls. Age is known to affect sensory and motor function and alter skin properties. For instance, discriminative touch is impaired with age^20^ and dexterity reduced^21,22^, with these changes interacting^23–25^. The average age of the participants (62.5 years) and the within-participant nature of the reporting in the present study make it likely that participants took their pre-chemotherapy status as a baseline thus minimising possible age effects in our data. Support for this view can be found in the results of a normative study of QLQ responses by Mols and colleagues^26^ in a non-patient sample (average age 53 years). For QLQ questions 1,3,5 and 7 (hand symptoms) the percentages reporting impairment in their study, averaged across the questions, was 8.7% compared to 55% for participants in the current study. Furthermore, the tabled values provided by Mols et al.^26^ showed that, for these questions, there was no difference in the average transformed CIPN scores between people in their 50’s (mean score of 3.8) and those in their 60’s (mean score of 3.9). While it is unlikely that age effects contributed significantly to the present findings, future studies may benefit from the inclusion of age-matched controls. Such inclusion would help distinguish between age-related and CIPN-specific effects on sensorimotor function and allow exploration of how aging and CIPN interact, facilitating the development of CIPN interventions.

A common issue in focus group research is determining whether saturation has been achieved^27^ or whether additional or larger focus groups would yield further themes. Our approach is based on deductive qualitative analysis^5^ with hand, task (buttoning, writing, jar opening) and sensorimotor control as preselected themes. So rather than saturation, in DQA an important issue is the degree of negative instances (counter evidence to preselected themes). In our case these might, for example, be taken as those cases where pain rather than numbness, was the disruptor of manual action. Such cases did occur but, rather than falsifying the preselected themes, they show the diversity of mechanisms at work potentially limiting manual action in CIPN.

Three questionnaires were used in the present study. Two (QLQ and PNQ) were pre-existing measurement tools in the literature. UBQ was a questionnaire developed by the authors specifically for the study, to extend reporting back over a longer timescale. Specifically, reporting was extended back to the period of chemotherapy treatment and, additionally, participants were able to comment in an open-ended manner on their experience of CIPN. UBQ was not a formally developed assessment tool with an underlying normative basis. In future research it would be useful to develop the questionnaire in this direction. It would also be advantageous to include questions focusing on specific CIPN-related problems identified in the present study, such as, with what implements, and under what circumstances, does dropping occur?

Questionnaire data, while capable of yielding valuable insights into participants’ experiences of CIPN, are prone to issues around subjective reporting, such as dependence on awareness, observation skills, recall abilities and response biases. To address these limitations, questionnaires might be supplemented with objective, quantitative measures of tactile impairment. For example, monofilament light pressure detection thresholds, as used by Molliasotis et al.^28^ and vibration detection thresholds, as used by Neilson and colleagues^29^, correlate with QLQ measures of CIPN. It is interesting to note some recent evidence from nerve conduction studies (NCS), which are sometimes regarded as the gold standard for diagnosing and monitoring peripheral neuropathies^30^. In this study, NCS did not correlate well with PROMs in CIPN^31^, and the authors suggested that NCS may not perform well in either discriminating clinically relevant CIPN or reflecting CIPN impact on patients. This potentially important result may need further study.

Generally, current approaches to objective measurement of CIPN effects on upper limb function focus on tests examining impairment in limited domains such, as light pressure or vibration detection. However, as the present study demonstrates, experiences of CIPN are not limited to sensory impairment but involve changes in sensorimotor function, impacting activities carried out in daily life. Objective quantification of CIPN might be enhanced by extending passive measures to include active touch, where the participant controls contact with the stimulus, such as discrimination of surface texture^32^, or object shape^33^. Touch feedback for action could also be assessed, for example, in terms of speed and kinematics, as with the Purdue pegboard test^34,35^. Or, if an instrumented manipulandum is available, assessment could be extended to use contact force as indicators of the use of touch in predictive and reactive control of grip^25,36^. Such measures will have the further advantage of potentially revealing underlying causal mechanisms whereas questionnaires, such as those used in the present study, are better suited to demonstrating impact of those changes.

## Conclusion

Participants’ responses to questionnaires and themes developed in focus groups clearly indicated the impact of CIPN-related touch impairment on a range of everyday manual activities. Examining the affected activities, together with participants’ reports of alterations in touch experience, have led to hypotheses about why some tasks are more affected than others.

An important element in coping with CIPN is an appreciation by patients and their clinicians of the nature of the disorder^7,15^. We suggest a theoretically based interpretation of the sensorimotor problems in CIPN. Sharing such understanding with those experiencing CIPN and their carers may contribute to an improved sense of involvement in symptom management. This, in turn, could help to empower people with CIPN to explore strategies to overcome the challenges of CIPN. We encourage the inclusion of objective, behavioural measures of action and perception in future CIPN studies and clinical trials to demonstrate the impact of impaired touch on sensorimotor control and improve evaluation of CIPN interventions.

## Supplementary Information

Below is the link to the electronic supplementary material.

Supplementary Material 1

## Data Availability

The questionnaire data that support the findings of this study are available in the main text and Supplementary Materials for this article. We can refer to, discuss, and/or paraphrase selected sections of the full transcripts on request from interested researchers, but the raw text cannot be made available due to privacy and ethical restrictions.
