# Supplementary material for: Touch and manual action in chemotherapy-induced peripheral neuropathy: a mixed-methods study
Source: Sci Rep. 2026 Mar 29;16:10689. doi: 10.1038/s41598-026-46039-2 (PMC13039469; doi:10.1038/s41598-026-46039-2)
Supplement: Supplementary file 1 — Supplementary Material 1 [file 41598_2026_46039_MOESM1_ESM.docx]

## Scientific Reports

**Discriminative touch for manual action in
chemotherapy induced peripheral neuropathy.**

Roberta D. Roberts^1^, Winnie Chua^1^, Ali Khatibi^1^, Nicholas P. Holmes^2^, Claire Palles^3,4^, Racha Kussaibati^5^, Alan M. Wing^1^.

^1^School of Psychology, University of Birmingham, Edgbaston, Birmingham, United Kingdom

^2^School of Sport, Exercise and Rehabilitation Sciences, University of Birmingham, Edgbaston, Birmingham, United Kingdom

^3^Department of Cancer and Genomic Sciences, University of Birmingham, Edgbaston, Birmingham, United Kingdom

^4^The National Institute for Health and Care Research (NIHR) Birmingham Biomedical Research Centre, United Kingdom

^5^ Department of Oncology, Heartlands Hospital, University Hospitals Birmingham NHS Foundation Trust, Birmingham, United Kingdom

Corresponding Author

Roberta D. Roberts
School of Psychology,
University of Birmingham,
Edgbaston,

Birmingham,
United Kingdom

Email: R.Roberts@bham.ac.uk

**Supplementary Materials**

| **(Table S1) The number of participants selecting each multiple choice option for questions in the University of Birmingham Questionnaire (UBQ).** | | | | |
| --- | --- | --- | --- | --- |
| **UBQ Questions** | **Number of Responses** | | | |
|  | **Yes** | **No** |  |  |
| **Item 2.** Did/do you have numbness and/or tingling as a result of chemotherapy? | 25 | 0 | - | - |
|  | **Hands** | **Feet** | **Both** |  |
| **Item 3a.** Does the numbness/tingling affect your Hands, Feet, Both,  Other body parts (please specify below) | 6 | 2 | 17 | - |
|  | **During chemotherapy** | | **After the end of chemotherapy** | |
| **Item 4.** When did the symptoms appear? | 18 | | 7 | |
|  | **Handling objects** | **Balance** | **Both** | **Other** |
| **Item 5.** Does the numbness/tingling affect your | 12 | 1 | 9 | 3 |
|  | **Getting worse** | **Getting better** | **Not changing** |  |
| **Item 6.** Is the numbness/tingling.... | 3 | 6 | 16 | - |
|  | **Yes** | **No** |  |  |
| **Item 7a.** Have you found any strategies or tricks that help reduce the impact of the numbness/ tingling on your activities? | 11 | 14 | - | - |
|  | **Yes** | **No** |  |  |
| **Item 9a.** Did you, or do you have, any other symptoms related to chemotherapy on your hands/feet as well as the numbness/tingling (e.g. damaged fingernails)? | 11 | 14 | - | - |

|  | | | | |
| --- | --- | --- | --- | --- |
| **(Table S2) The number of participants selecting each option in the European Organization for Research and Treatment of Cancer Quality of Life Questionnaire (QLQ).** | | | | |
| **QLQ Questions** | **Number of Responses** | | | |
|  | **Not at all (1)** | **A little**  **(2)** | **Quite a bit**  **(3)** | **Very much**  **(4)** |
| 1. Did you have tingling fingers or hands | 8 | 12 | 2 | 3 |
| 2. Did you have tingling toes or feet? | 10 | 6 | 4 | 5 |
| 3. Did you have numbness in your fingers or hands? | 9 | 10 | 2 | 3 |
| 4. Did you have numbness in your toes or feet? | 8 | 7 | 7 | 3 |
| 5. Did you have shooting or burning pain in your fingers or hands? | 18 | 3 | 1 | 3 |
| 6. Did you have shooting or burning pain in your toes or feet? | 17 | 6 | 0 | 2 |
| 7. Did you have cramps in your hands? | 18 | 4 | 1 | 2 |
| 8. Did you have cramps in your feet? | 14 | 9 | 1 | 1 |
| 9. Did you have problems standing or walking because of difficulty feeling the ground under your feet? | 20 | 3 | 1 | 1 |
| 10. Did you have difficulty distinguishing between hot and cold water? | 14 | 4 | 6 | 1 |
| 11. Did you have a problem holding a pen, which made writing difficult? | 13 | 10 | 1 | 1 |
| 12. Did you have difficulty manipulating small objects with your fingers (for example, fastening small buttons) | 7 | 12 | 2 | 4 |
| 13. Did you have difficulty opening a jar or bottle because of weakness in your hands? | 12 | 6 | 5 | 2 |
| 14. Did you have difficulty walking because your feet dropped downwards? | 24 | 0 | 0 | 1 |
| 15. Did you have difficulty climbing stairs or getting up out of a chair because of weakness in your legs? | 20 | 3 | 1 | 1 |
| 16. Were you dizzy when standing up from a sitting or lying position? | 16 | 8 | 0 | 1 |
| 17. Did you have blurred vision? | 21 | 3 | 0 | 0 |
| 18. Did you have difficulty hearing? | 19 | 2 | 2 | 1 |
| Please answer the following question only if you drive a car  19.Did you have difficulty using the pedals? | 18 | 3 | 0 | 1 |
| Please answer the following question only if you are a man:  20. Did you have difficulty getting or maintaining an erection? | 3 | 3 | 0 | 1 |

| **(Table S3) The number of participants selecting each option in the Patient Neurotoxicity Questionnaire (PNQ)** | | | | | |
| --- | --- | --- | --- | --- | --- |
| **Item 1.** | I have no numbness, pain or tingling in my hands or feet. | I have mild tingling, pain or numbness in my hands or feet. This does not  interfere with my activities of daily living. | I have moderate tingling, pain or numbness in my hands or feet. This does not  interfere with my activities of daily living. | * I have moderate to severe, tingling, pain or numbness in my hands or feet.  This interferes with my activities of daily living. | * I have severe tingling, pain or numbness in my hands or feet. It completely  prevents me from doing most activities of daily living. |
| **Number  Selected** | 1 | 6 | 11 | 6 | 1 |
|  | | | | | |
| **Item 2.** | I have no weakness in my arms or legs. | I have mild weakness in my arms or legs. This does not interfere with my activities of daily living. | I have moderate weakness in my arms or legs. This does not interfere with my activities of daily living. | * I have moderate to severe weakness in my arms or legs. This interferes with my activities of daily living. | * I have severe weakness in my arms or legs. It completely prevents me from doing most activities of daily living. |
|  | 13 | 7 | 2 | 2 | 1 |

| **Item 3.** | | | |
| --- | --- | --- | --- |
| If you have selected options marked with an asterisk (*) above, please indicate by selecting options or describing in the space provided below, which activity/activities have been interfered with as a result of chemotherapy. | | | |
| Button Clothes | **11** | Climb stairs | **3** |
| Use a knife | **4** | Type on a keyboard | **3** |
| Use a fork | **4** | Write | **5** |
| Use a spoon | **3** | Walk | **3** |
| Other eating utensils, etc | **1** | Put on jewellery | **7** |
| Open doors | **2** | Knit | **0** |
| Put in or remove contact lenses | **1** | Sew | **4** |
| Dial or use telephone | **4** | Work | **1** |
| Operation of remote control | **1** | Tie shoelaces | **6** |
| Fasten buckles | **7** | Drive | **2** |
| Sleep | **7** | Using touchscreens (e.g. smartphones, iPad/tablet) | **6** |

| **(Table S4) University of Birmingham Questionnaire (UBQ) on symptoms of CIPN**  This first questionnaire below is concerned with understanding if you have ever had symptoms of CIPN, how they have affected touch in your hands and/or feet, what impact this has had on your activities, and what helps to reduce the impact. | | | | |
| --- | --- | --- | --- | --- |
| 1. | When did you finish your chemotherapy? | |  |  |
| 2. | Did/do you have numbness and/or tingling as a result of chemotherapy?  If No please skip to the next questionnaire by scrolling to the end of this page and clicking Next. | | | |
|  | Yes | No |  |  |
| 3a. | Does the numbness/tingling affect your | | | |
|  | Hands |  |  |  |
|  | Feet |  |  |  |
|  | Both |  |  |  |
|  | Other body parts (please specify below) | |  |  |
|  |  |  |  |  |
|  |  |  |  |  |
| 3b. | How much of your body part is affected by the numbness/tingling? | | |  |
|  |  |  |  |  |
| 4. | When did the symptoms appear? | | |  |
|  | During chemotherapy | |  |  |
|  | After the end of chemotherapy | |  |  |
|  |  |  |  |  |
| 5a. | Does the numbness/tingling affect your | | |  |
|  | Handling objects |  |  |  |
|  | Balance |  |  |  |
|  | Both |  |  |  |
|  | Other |  |  |  |
|  |  |  |  |  |
| 5b. | How does the numbness/tingling affect these activities? | | |  |
|  |  |  |  |  |
|  |  |  |  |  |
| 6. | Is the numbness/tingling.... | |  |  |
|  | Getting worse |  |  |  |
|  | Getting better |  |  |  |
|  | Not changing |  |  |  |
|  |  |  |  |  |
| 7a. | Have you found anything that helps reduce the feeling of numbness/tingling? | | | |
|  |  |  |  |  |
| 7b. | Please describe what helps. | |  |  |
|  |  |  |  |  |
|  |  |  |  |  |
|  |  |  |  |  |
| 8a. | Have you found any strategies or tricks that help reduce the impact of the numbness/tingling on your activities? | | | |
|  | Yes | No |  |  |
|  |  |  |  |  |
| 8b. | Please describe these strategies. | |  |  |
|  |  | |  |  |
| 9a. | Did you, or do you have, any other symptoms related to chemotherapy on your hands/feet as well as the numbness/tingling (e.g. damaged fingernails)? | | | |
|  | Yes | No |  |  |
|  |  |  |  |  |
| 9b. | Please describe the other symptoms. | | | |
|  |  | | | |
|  |  | | | |
|  |  | | | |

**(Table S5) Focus group statements relating to sensory experience: foot numbness and tingling and pain. The statements relate to numbness, tingling and to pain. Comments under numbness and tingling include reductions in CIPN symptoms. Under the pain heading, statements include reference to pain interfering with tasks in which fingertip pressure is required**.

| **Group** | **ID** | **Time** | **Statement** |
| --- | --- | --- | --- |
|  |  |  | **Numbness and tingling** |
| A | 9 | 52:47 | My feet are much more sensitive to what's under the sole. in fairly cold water … initially it felt as if I were walking through seaweed … mixed sensations around my ankles, which eventually I adapted to and got used to. |
| A | 9 | 53:57 | … it's much more the level of the feet, and you know, and I have tingling when I'm lying in bed. |
| B | 11 | 25:46 | I've had numbness in my toes .. ever since I had chemo (*6 years ago*) ... occasionally I'm aware of balance not being right. But it's ... manageable. But it's been there for well, for 6 years. |
| B | 10 | 26:37 | trip quite a lot at the beginning. and I did fall down 3 stairs as well in in the house once, and I've taken avoiding strategies for that. Really, I walk more slowly, and that has solved the tripping problem. I never wear shoes with any sort of heel on them, because it was a heel I caught coming down the stairs, and I always hold a rail, either in the house coming down the stairs or when I'm out. |
|  |  |  | **Pain** |
| A | 25 | 51:31 | It's the pain for me in the cold. … I've got to have my foot on the clutch for any length of time. … The pressure brings on too much pain to concentrate on driving. So at least with an automatic, you can have one foot free. |
| A | 25 | 52:19 | So I never stand still either. So if I'm in a queue for something, I don't stand still. I can't, because the pain is too great. |
| B | 24 | 23:24 | My left foot … is affected and has been ever since I started chemotherapy (*3 years ago*)… a burning sensation .. when stepping up, down stairs or steps. I'm very conscious, as I can't always feel the ground properly. |
| B | 10 | 28:49 | I wear bed socks, thick bed socks every night in bed because I've got allodynia, which is this sort of intensive sensitivity of my feet and that helps. |
